# METTL3 boosts mitochondrial fission and induces cardiac fibrosis after ischemia/reperfusion injury

**DOI:** 10.7150/ijbs.87535

**Published:** 2024-01-01

**Authors:** Li Ma, Xing Chang, Jing Gao, Ying Zhang, Ye Chen, Hao Zhou, Na Zhou, Na Du, Jiamin Li, Jiachen Bi, Ziyue Chen, Xinxin Chen, Qingyong He

**Affiliations:** 1 Heart Center, Guangdong Provincial Key Laboratory of Research in Structural Birth Defect Disease, Guangzhou Women and Children's Medical Center, Guangzhou Medical University, Guangzhou 510623, China.; 2 Guang'anmen Hospital, China Academy of Chinese Medical Sciences, Beijing, 100053, China.; 3 Senior Department of Cardiology, The Sixth Medical Center of People's Liberation Army General Hospital, Beijing 100048, China.

**Keywords:** METTL3, DNA-PKcs, Fis1, mitochondrial fission, cardiac ischemia-reperfusion injury.

## Abstract

METTL3, an RNA methyltransferase enzyme, exerts therapeutic effects on various cardiovascular diseases. Myocardial ischemia-reperfusion injury (MIRI) and subsequently cardiac fibrosis is linked to acute cardiomyocyte death or dysfunction induced by mitochondrial damage, particularly mitochondrial fission. Our research aims to elucidate the potential mechanisms underlying the therapeutic actions of METTL3 in MIRI, with focus on mitochondrial fission. When compared with *Mettl3^flox^* mice subjected to MIRI, Mettl3 cardiomyocyte knockout (*Mettl3^Cko^*) mice have reduced infarct size, decreased serum levels of myocardial injury-related factors, limited cardiac fibrosis, and preserved myocardial ultrastructure and contractile/relaxation capacity. The cardioprotective actions of *Mettl3* knockout were associated with reduced inflammatory responses, decreased myocardial neutrophil infiltration, and suppression of cardiomyocyte death. Through signaling pathway validation experiments and assays in cultured HL-1 cardiomyocytes exposed to hypoxia/reoxygenation, we confirmed that *Mettl3* deficiency interfere with DNA-PKcs phosphorylation, thereby blocking the downstream activation of Fis1 and preventing pathological mitochondrial fission. In conclusion, this study confirms that inhibition of METTL3 can alleviate myocardial cardiac fibrosis inflammation and prevent cardiomyocyte death under reperfusion injury conditions by disrupting DNA-PKcs/Fis1-dependent mitochondrial fission, ultimately improving cardiac function. These findings suggest new approaches for clinical intervention in patients with MIRI.

## Introduction

Myocardial ischemia-reperfusion injury (MIRI) refers to a pathological condition characterized by a temporary reduction or interruption of blood flow to the myocardium, followed by rapid reperfusion of the ischemic area 1-3. More than 50% of patients with myocardial infarction experience MIRI, either before, during, or after undergoing percutaneous coronary intervention (PCI) 4, 5. The occurrence of MIRI renders the heart muscle vulnerable due to inadequate energy supply and accumulation of metabolic waste 6, 7. MIRI has the potential to exacerbate the size of the infarction and increase mortality in patients undergoing PCI 8-10. Unfortunately, there are currently no effective pharmacological approaches to mitigate the numerous myocardial abnormalities caused by reperfusion possibly due to the complex mechanisms underlying MIRI-mediated myocardial damage and cardiac fibrosis.

METTL3, an RNA methyltransferase enzyme, exerts a crucial role in gene expression regulation by methylating RNA molecules through catalysis. While METTL3 is primarily recognized for its involvement in RNA modification and processing, recent investigations have indicated its potential contribution to cardiovascular diseases, such as atherosclerosis. METTL3-mediated RNA methylation has been linked to the development and progression of atherosclerosis 11, affecting the stability and function of specific mRNAs related to endothelial cell function, lipid metabolism, and inflammation 12, 13. Furthermore, METTL3 has been implicated in cardiac hypertrophy 14, where its m6A RNA methylation mechanism influences the expression of key genes associated with myocardial remodeling, fibrosis, and contractile function 15-17. Dysregulation of METTL3-mediated RNA methylation has also been observed in heart failure models 16, 18, 19, impacting the expression of genes involved in cardiac function, calcium handling, and energy metabolism. Additionally, METTL3 has been found to modulate the function of vascular smooth muscle cells (VSMCs) by regulating the expression of genes involved in vascular remodeling, contractility, and inflammation 20, 21. The dysregulation of METTL3-mediated RNA methylation in VSMCs has been associated with vascular diseases, including hypertension 22 and vascular remodeling 23. It is worth noting that our understanding of METTL3's role in cardiovascular diseases is still developing, and further investigations are needed to elucidate the underlying mechanisms and potential therapeutic implications. In this study, our objective was to investigate whether suppression of METTL3 can reduce acute MIRI in mice.

Although several cellular alterations have been shown to underlie MIRI symptomatology, evidence suggests that therapeutic targeting of mitochondrial dysfunction is a promising way to reduce its sequelae 24-26. Our previous studies highlighted the contribution of mitochondria-dependent metabolic imbalance to cardiomyocyte dysfunction and death 27-29. Following studies further reported the indispensable roles played by mitochondrial calcium overload 30 and reactive oxygen species (ROS) overproduction 31 in aggravating MIRI-related myocardial contraction/relaxation deficits. Our recent reports further highlighted the functional importance of disrupted mitochondrial dynamics, especially mitochondrial fission, in the MIRI setting 32-34. Our published findings are in good accordance with other reports which defined mitochondrial fission as a contributor to MIRI 35-37. Considering the complex involvement of mitochondrial fission in triggering cardiomyocyte death and myocardial dysfunction, it is of interest to evaluate whether deletion of METTL3 reduces MIRI through suppressing mitochondrial fission. Importantly, recent studies have reported that mitochondrial fission during the myocardial reperfusion phase is tightly controlled by DNA-PKcs/Fis1 signaling 38, 39. Therefore, another task of this investigation was to assess whether *Mettl3* deficiency repressed MIRI-induced mitochondrial fission through regulation of the DNA-PKcs/Fis1 pathway.

## Results

### *Mettl*3 knockout reduces reperfusion-induced cardiac fibrosis and myocardial dysfunction

**Figure 1:**
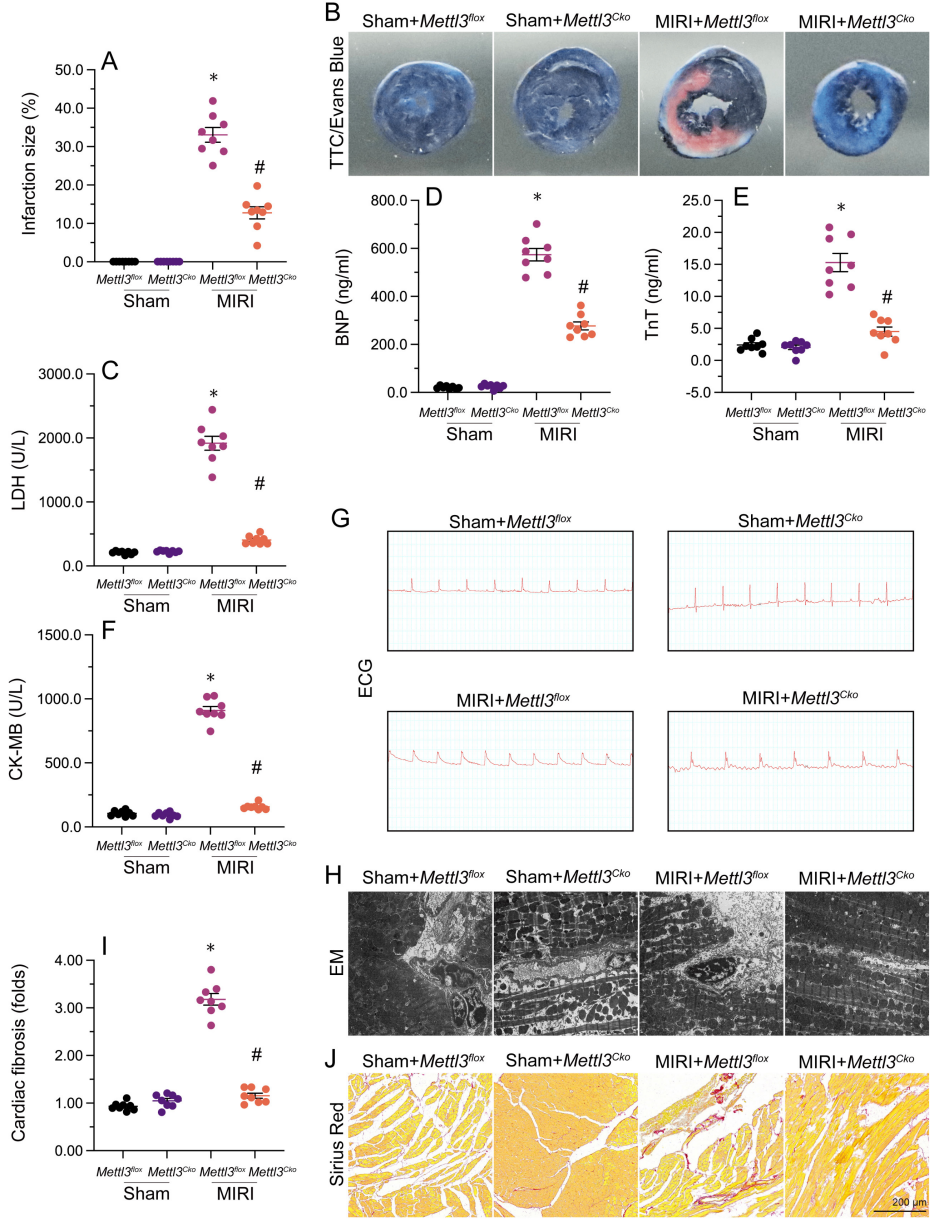
***Mettl3* knockdown reduces reperfusion-related myocardial dysfunction.** *Mettl3^flox^* and *Mettl3^Cko^* mice were subjected to 30-min ischemia via temporary LAD ligation, followed by 6-h reperfusion to induce MIRI. **(A**, **B)** TTC and Evans blue stainings were used to examine the extent of myocardial infarction.** (C-F)** ELISA was used to measure serum concentrations of myocardial injury biomarkers, including Troponin T (TnT), creatine kinase MB (CK-MB), lactate dehydrogenase (LDH), and brain natriuretic peptide (BNP). **(G)** Representative ECG signals from mice subjected to MIRI. **(H)** Ultrastructural analysis of the myocardium using EM. **(I-J)** Sirius Red staining of cardiac fibrosis. *p<0.05 vs. sham group, #p<0.05 vs. MIRI group.

To delineate the cardioprotective impact of *Mettl3* deficiency on MIRI, we employed TTC and Evans blue staining to depict the extent of MIRI in mice subjected to temporary LAD ligation and subsequent coronary reperfusion. Results revealed that loss of *Mettl3* significantly reduced infarction size compared to untreated mice (Figure 1A and 1B). Additionally, we investigated the levels of myocardial injury biomarkers, including Troponin T (TnT), creatine kinase MB (CK-MB), lactate dehydrogenase (LDH), and brain natriuretic peptide (BNP), in mouse serum. Following MIRI, these biomarkers were markedly upregulated, but loss of *Mettl3* effectively reduced their levels (Figure 1C-1F).

Furthermore, electrocardiogram (ECG) analysis demonstrated that MIRI was characterized by elevated ST segments and T-wave inversion, whereas the above alterations were largely alleviated in *Mettl3*-deleted mice (Figure 1G). Moreover, after MIRI induction, electron microscopy (EM) revealed characteristic myocardial edema and myofibrillar disarray, accompanied by cardiomyocyte rupture and mitochondrial structural damage, in untreated mice. Remarkably, ablation of *Mettl3* largely preserved the histological integrity of the heart (Figure 1H). After four weeks following myocardial ischemia-reperfusion injury (MIRI), the hearts of mice were subjected to Sirius Red staining to assess the presence of cardiac fibrosis (Figure 1I-J). The findings revealed that MIRI led to significant myocardial fibrosis and cardiac remodeling. However, in mice with *Mettl3* deletion, these alterations were not observed, indicating that *Mettl3* deletion may protect against MIRI-induced cardiac fibrosis. These results demonstrated that *Mettl3* deficiency preserves cardiac histology and attenuates myocardial dysfunction induced by MIRI.

### Knockout of *Mettl3* maintains heart function following MIRI

**Figure 2:**
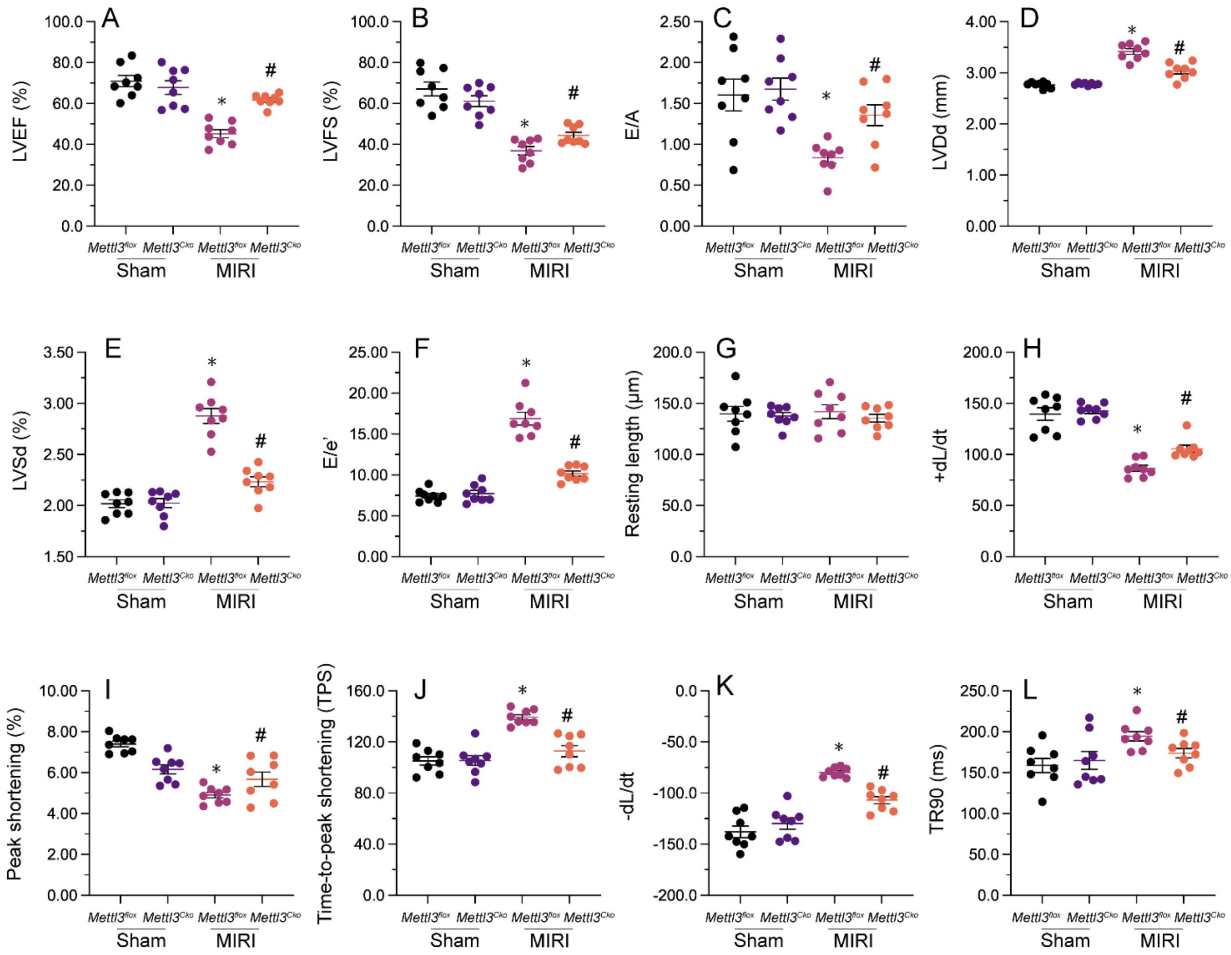
***Mettl3* deletion maintains heart function following cardiac I/R. (A-F)** Echocardiography was utilized to assess MIRI-related changes in heart function in *Mettl3^flox^* and *Mettl3^Cko^* mice. **(G-L)** Analysis of the contractile properties of cardiomyocytes isolated from *Mettl3^flox^* and *Mettl3^Cko^* mice. *p<0.05 vs. sham group, #p<0.05 vs. MIRI group.

Echocardiography was utilized to assess heart function after MIRI. As shown in Figure 2A-2F, MIRI was linked to significant decreases in left ventricular ejection fraction (LVEF) and fractional shortening (LVFS), along with increases in left ventricular end-diastolic diameter (LVDd) and end-systolic diameter (LVSd). Additionally, there was an increase in the E/e' ratio and a decrease in the E/A ratio, indicating impaired heart relaxation capacity. Remarkably, deletion of *Mettl3* in cardiomyocytes markedly reversed the reductions in LVEF/LVFS and normalized LVDd/LVSd, while restoring the E/A and E/e' ratios to physiological levels (Figure 2A-2F).

Next, we isolated cardiomyocytes from MIRI-exposed mice to investigate their contractile properties. Initially, we noted that MIRI had no impact on cardiomyocyte length, regardless of *Mettl3* deficiency. However, as shown in Figure 2G-2L, peak shortening (PS) and maximal shortening velocity (+dL/dt) were reduced upon MIRI, and these parameters were significantly restored in cardiomyocytes derived from *Mettl3^Cko^* mice. In addition, exposure to MIRI led to increased time-to-peak shortening (TPS), reduced maximal velocity of relengthening (-dL/dt), and prolongation of the time-to-90% relengthening (TR90). Interestingly, MIRI failed to impair TPS, -dL/dt, and TR90 in cardiomyocytes from *Mettl3^Cko^*-treated animals (Figure 2G-2L). These findings confirmed that *Mettl3* knockout preserves heart function in mice subjected to MIRI.

### *Mettl3 deletion* alleviates myocardial inflammation and cardiomyocyte death caused by MIRI

**Figure 3:**
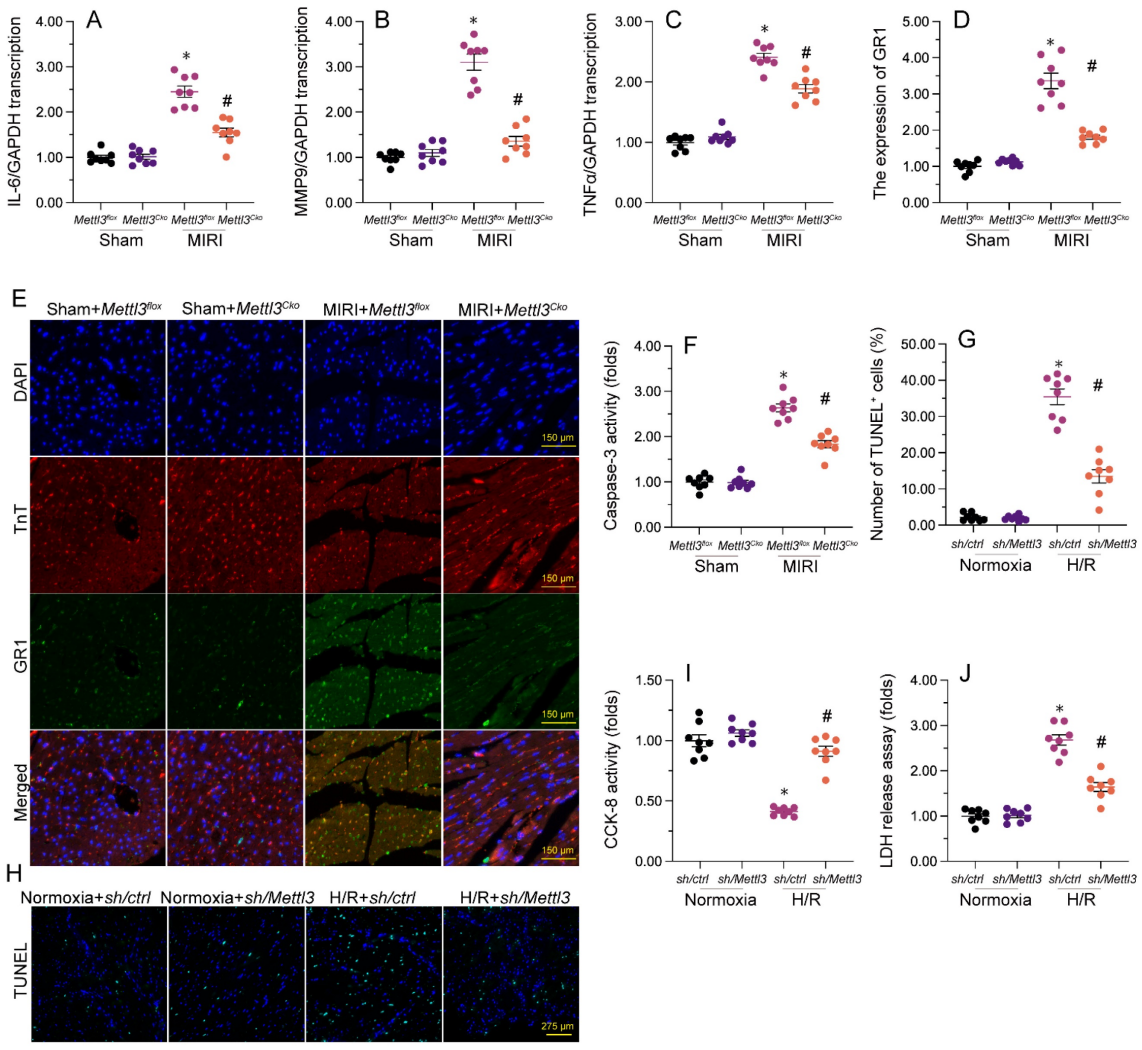
***Mettl3* deficiency reduces myocardial inflammation and cardiomyocyte death after MIRI induction. (A-C)** RT-qPCR analysis of the transcription of pro-inflammatory genes, including IL-6, MM9, and TNFα, in mouse cardiac tissue. **(D-E)** Gr-1 immunofluorescence was conducted in heart tissues to determine neutrophil infiltration rates. A TnT antibody was used to stain myofibrils and DAPI was used for nuclear counterstaining. **(F)** ELISA was used to observe changes in caspase-3 activity in cultured HL-1 cardiomyocytes exposed to hypoxia/reoxygenation (H/R). **(G, H)** TUNEL staining was used to detect apoptosis in H/R-treated HL-1 cells. **(I)** Results of CCK-8 assays, used to determine the effect of *Mettl3* deletion on the viability of H/R-exposed HL-1 cells. **(J)** The concentration of LDH in cultured media from HL-1 cells was determined by ELISA. *p<0.05 vs. sham group or Ctrl group, #p<0.05 vs. MIRI group or H/R group.

Previous studies have established that myocardial inflammation and cardiomyocyte death are primary pathological changes in MIRI. RT-qPCR analysis of heart tissues revealed that MIRI induction led to significant upregulation of the transcription of pro-inflammatory genes, as illustrated by elevated transcription of IL-6, MMP9, and TNFα (Figure 3A-3C). However, consistent with anti-inflammatory properties for *Mettl3* deletion during MIRI, in samples from *Mettl3^Cko^* mice, the expression of these markers was maintained at near normal levels (Figure 3A-3C). Additionally, immunofluorescence staining demonstrated that the relative immunofluorescence intensity of GR-1, a surface marker of neutrophils, was significantly increased in cardiac tissue after I/R induction, but undetectable instead following *Mettl3* deletion (Figure 3D and 3E). These findings suggest that *Mettl3* knockout effectively suppresses the infiltration of inflammatory cells into heart muscle during MIRI.

To evaluate whether *Mettl3* deletion protects against cardiomyocyte death induced by MIRI, caspase-3 activity was examined in cardiac tissue using ELISA. Results revealed significant induction of caspase-3 activity upon MIRI, and partial attenuation of this effect after deletion of *Mettl3* (Figure 3F). Furthermore, TUNEL experiments indicated that MIRI resulted in substantial cardiomyocyte death (>30%), whereas this rate was reduced to approximately 13% after deletion of *Mettl3* (Figure 3G and 3H). Consistently, CCK-8 (Figure 3I) and LDH release (Figure 3J) assays in HL-1 cardiomyocytes *in vitro* further demonstrated the pro-survival effect of *Mettl3* deletion when applied prior to hypoxia/reoxygenation (H/R) injury. These data thus support anti-inflammatory and anti-apoptotic actions of *Mettl3* deletion during MIRI.

### *Mettl3* knockdown attenuates H/R-induced mitochondrial abnormalities in cardiomyocytes

**Figure 4:**
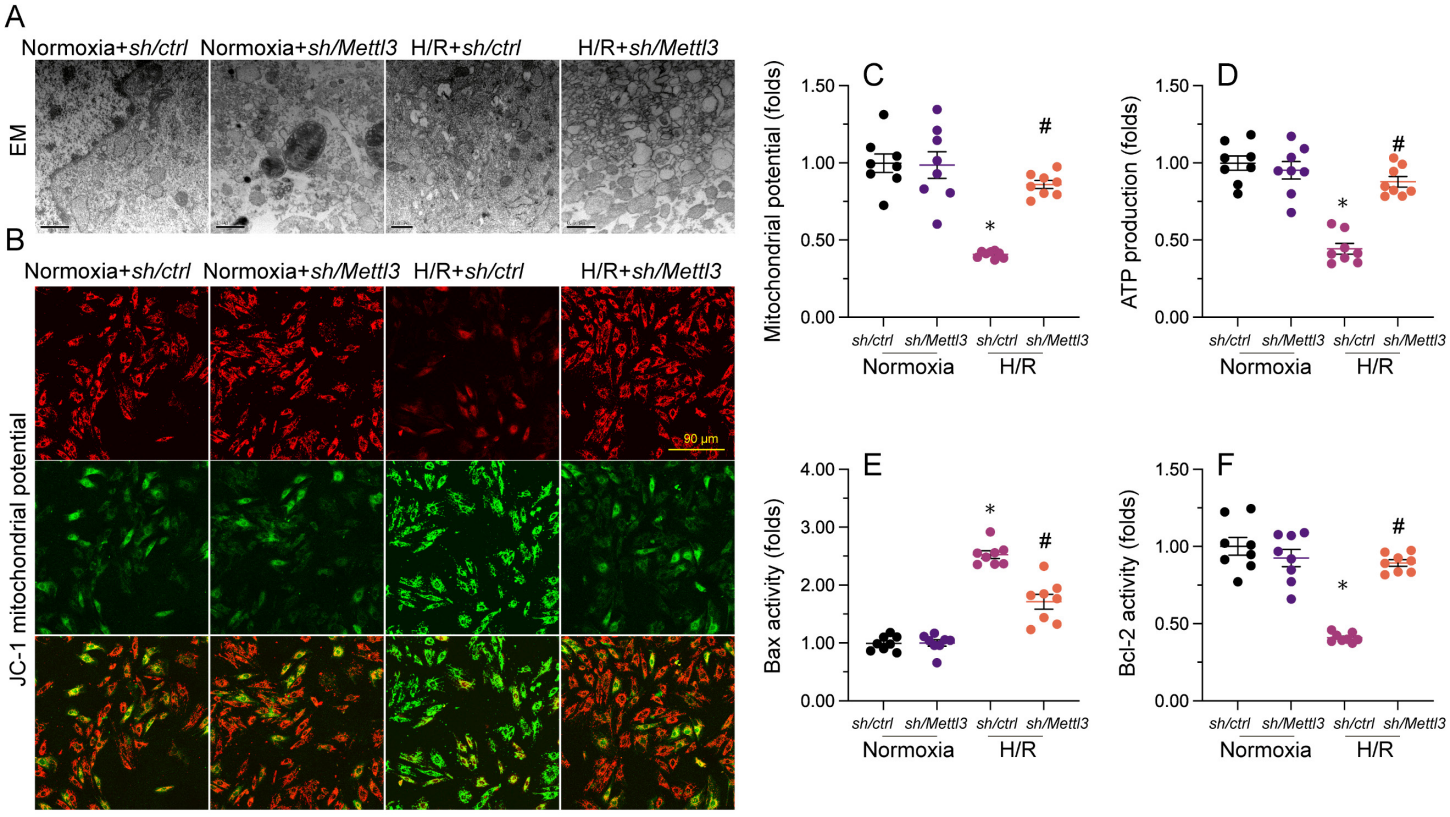
***Mettl3* deletion attenuates mitochondrial abnormalities in cardiomyocytes exposed to H/R injury. (A)**. EM was used to observe ultrastructural changes in mitochondria from H/R-treated HL-1 cells. **(B, C)** Mitochondrial membrane potential was evaluated in HL-1 cells loaded with JC-1 by quantification of the red-to-green fluorescence ratio. **(D)** Quantification of ATP production by ELISA in HL-1 cells. **(E, F)**. The activities of Bax and Bcl-2 in HL-1 cells were measured through ELISA. *p<0.05 vs. Ctrl group, #p<0.05 vs. H/R group.

Mitochondrial dysfunction represents a primary alteration associated with cardiomyocyte damage during MIRI. Therefore, we investigated whether *Mettl3* deletion could protect mitochondrial function in HL-1 cardiomyocytes upon H/R challenge. Ultrastructural analysis of mitochondria using EM illuminated that H/R caused small and round mitochondria (Figure 4A), accompanied by mitochondrial cristae remodeling and rupture. However, deletion of *Mettl3* significantly preserved mitochondrial crista integrity and partially maintained mitochondrial structure (Figure 4A). In addition to structural abnormalities, mitochondrial membrane potential was reduced upon H/R; this impact of which was counteracted by *Mettl3* deletion (Figure 4B and 4C). Furthermore, the concentration of ATP in cultured HL-1 cells was markedly decreased after H/R exposure, but was maintained at baseline levels upon *Mettl3* deletion (Figure 4D). Considering the crucial role of mitochondria in triggering cardiomyocyte death, we investigated whether the anti-apoptotic action of *Mettl3* deletion is mediated by suppression of the mitochondria-dependent cell death pathway. ELISA demonstrated that H/R increased Bax activity and reduced Bcl-2 activity in HL-1 cells. However, *Mettl3* deletion maintained the Bax/Bcl-2 balance (Figure 4E and 4F). These results support our hypothesis that *Mettl3* deficiency preserves mitochondrial function during MIRI.

### *Mettl3* deletion inhibits I/R-mediated mitochondrial fission in the mouse heart

**Figure 5:**
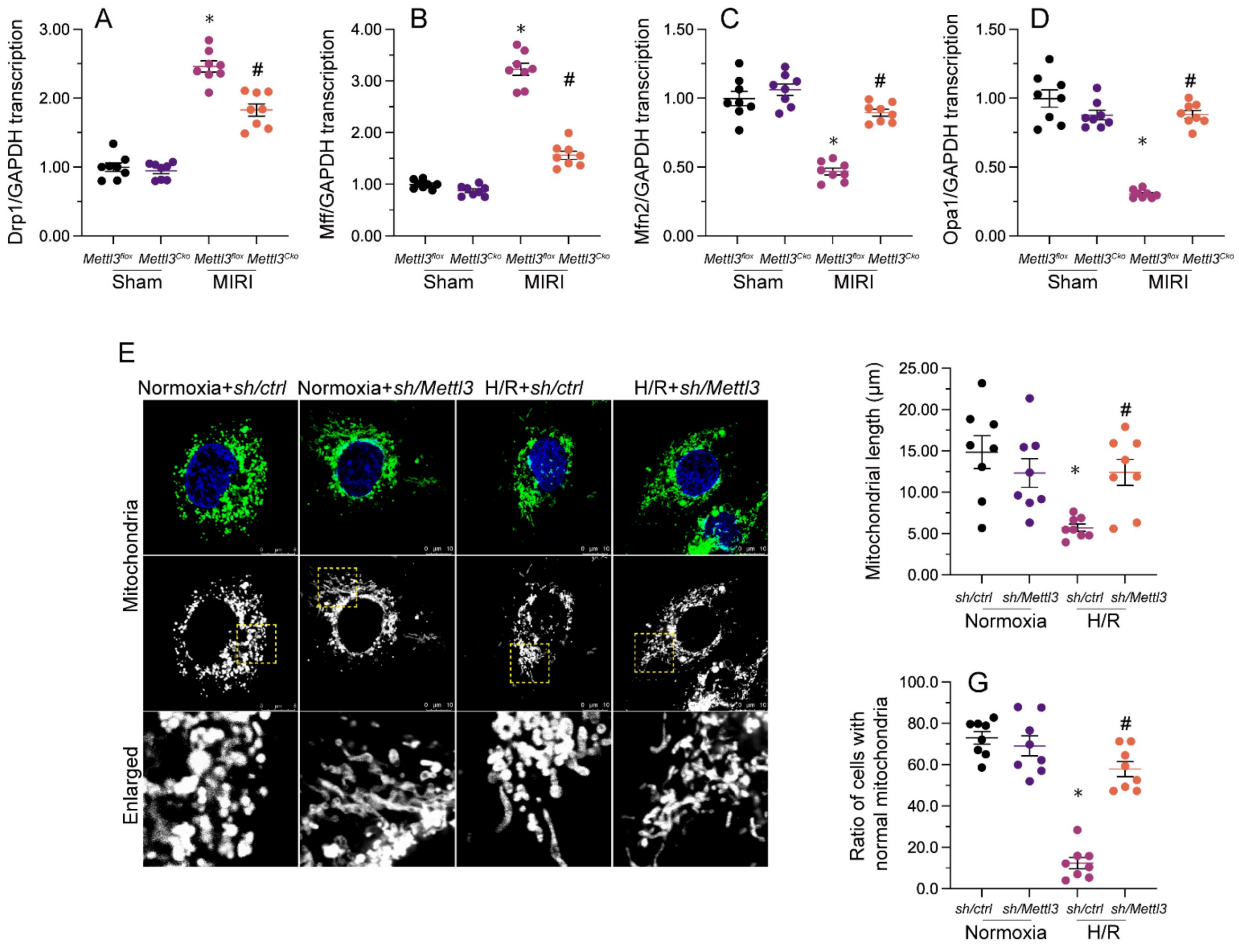
***Mettl3* ablation attenuates I/R- and H/R-mediated mitochondrial fission in cardiomyocytes. (A-D)** RT-qPCR-based transcriptional analysis of Drp1, Mff, Mfn2, and Opa1 expression in heart tissues. **(E-G)** Mitochondrial immunofluorescence was used to monitor mitochondrial fission. Mitochondrial length as well as the ratio of fragmented to tubular mitochondria were recorded. *p<0.05 vs. sham group or Ctrl group, #p<0.05 vs. MIRI group or H/R group.

Our previous studies demonstrated that inhibiting mitochondrial fission effectively reduces reperfusion-mediated mitochondrial dysfunction and preserves heart output 33, 38, 40. When relative to the sham-operated control group, a significant increase in cardiac transcription of mitochondrial fission-related genes, i.e. Drp1 and Mff, was detected following MIRI induction (Figure 5A-5D). Conversely, mRNA levels of mitochondrial fusion biomarkers, namely Mfn2 and Opa1, were substantially downregulated (Figure 5A-5D). Consistent with inhibition of mitochondrial fission, these expression trends were reversed upon *Mettl3* ablation (Figure 5A-5D). Meanwhile, investigations in HL-1 cells elucidated that H/R treatment promoted mitochondrial fission, evidenced by an increased ratio of fragmented to tubular mitochondria and decreased mitochondrial length (Figure 5E-5G), compared to control cells. However, consistent with the above *in vivo* findings, mitochondrial fragmentation was inhibited, while mitochondrial length was restored, following *Mettl3* knockdown treatment (Figure 5E-5G). These findings indicated that *Mettl3* deletion prevents mitochondrial fission in the reperfused heart.

### *Mettl3* knockout interacts directly with DNA-PKcs and prevents I/R-mediated DNA-PKcs phosphorylation and activation

**Figure 6:**
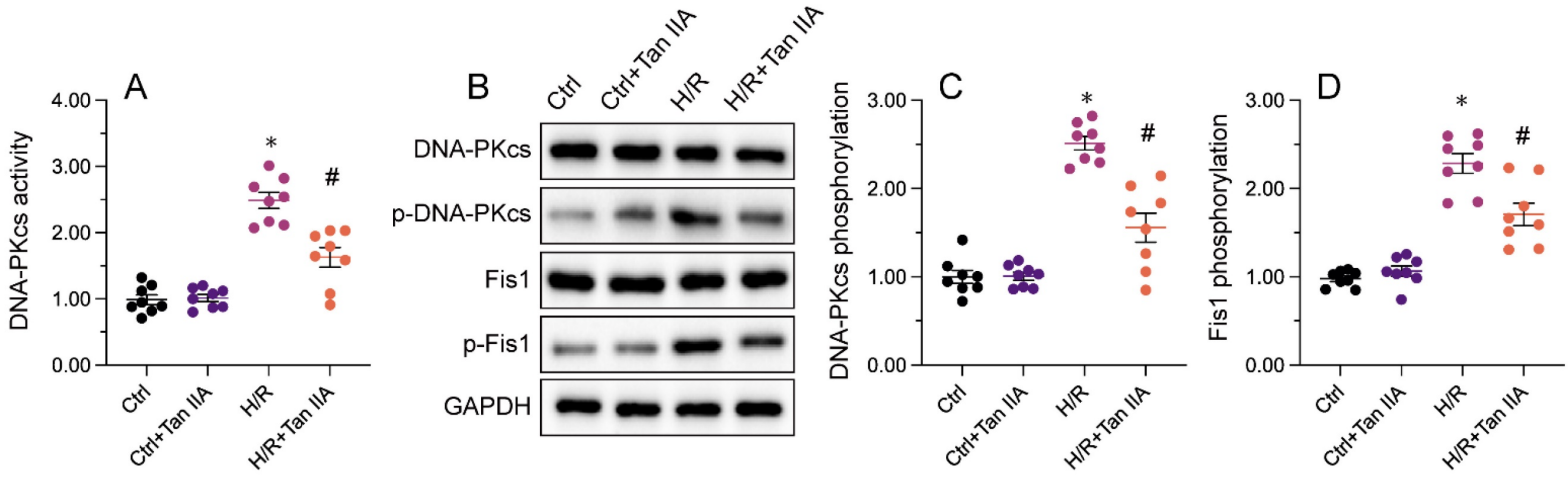
***Mettl3* deficiency prevents I/R-mediated DNA-PKcs phosphorylation and activation. (A)** ELISA was used to assess the activity of DNA-PKcs in HL-1 cells exposed to H/R injury. **(B-D)** Western blots were conducted to analyze DNA-PKcs and Fis1 phosphorylation status in H/R-treated HL-1 cells. *p<0.05 vs. Ctrl group, #p<0.05 vs. H/R group.

Recent evidence has shown that DNA-PKcs serves as a novel upstream regulator of mitochondrial fission in MIRI by facilitating Fis1 phosphorylation 38, 39. Considering the inhibitory impact of *Mettl3* deficiency on mitochondrial fission, we investigated whether *Mettl3* ablation blocks mitochondrial fission through regulation of DNA-PKcs. To assess the outcome of the Mettl3-DNA-PKcs interaction, ELISA was utilized to analyze the activity of DNA-PKcs. Results indicated a significant elevation in DNA-PKcs activity in HL-1 cells following H/R treatment, which was effectively reversed by *sh*/*Mettl3* (Figure 6A). Previous studies have indicated that DNA-PKcs activation is primarily reliant on post-transcriptional phosphorylation 31, 41. Consistently, western blots revealed that *Mettl3* deletion inhibited DNA-PKcs phosphorylation induced by H/R (figure 6B-6D). In addition, H/R-mediated phosphorylation of Fis1, a downstream effector of DNA-PKcs, was also significantly inhibited by *Mettl3* knockdown (Figure 6B-6D). These findings demonstrated that Mettl3 interacts directly with DNA-PKcs, thereby impeding H/R-activated DNA-PKcs through suppression of DNA-PKcs post-transcriptional phosphorylation.

### Loss of *Mettl3* improves mitochondrial performance and cardiomyocyte viability through interrupting the DNA-PKcs/Fis1/mitochondrial fission pathway

**Figure 7:**
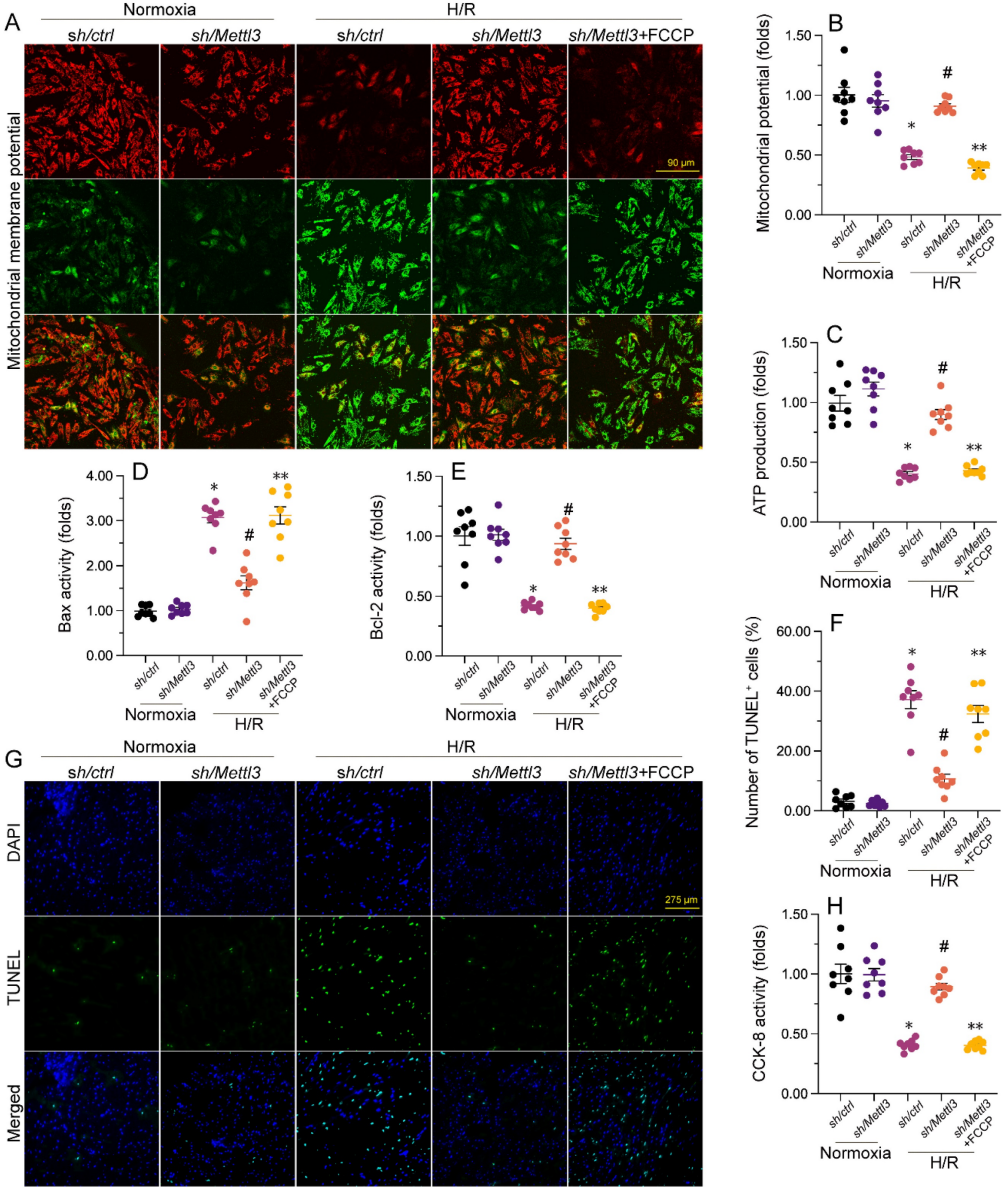
***Mettl3* deficiency improves mitochondrial performance and cardiomyocyte viability through interrupting the DNA-PKcs/Fis1/mitochondrial fission pathway.** FCCP was added to cultured HL-1 cells 30 min before H/R exposure to stimulate mitochondrial fission. **(A**, **B)** Mitochondrial membrane potential was measured in HL-1 cells loaded with the JC-1 probe by quantifying the relative red-to-green fluorescence ratio. **(C)** ATP production was measured via ELISA in cultured HL-1 cells. **(D**, **E)** The activities of Bax and Bcl-2 were measured through ELISA in cultured HL-1 cells. **(F**, **G)**. TUNEL staining was used to estimate apoptosis in cultured HL-1 cells. **(H)** Results of CCK-8 assays, conducted to quantify HL-1 cell viability. *p<0.05 vs. Ctrl group, #p<0.05 vs. H/R group, **p<0.05 vs. H/R+*sh/Mettl3*+FCCP group.

To further elucidate whether *Mettl3* deletion regulates mitochondrial function and cardiomyocyte viability through the DNA-PKcs/Fis1/mitochondrial fission cascades in the presence of H/R injury, FCCP, an activator of mitochondrial fission, was added to cultured HL-1 cells prior to H/R exposure. Interestingly, pre-incubation with FCCP abrogated the stabilizing action of *Mettl3* deletion on mitochondrial membrane potential (Figure 7A and 7B). Furthermore, ELISA results revealed that in H/R-exposed cells treated with FCCP, *Mettl3* ablation failed to both sustain ATP synthesis (Figure 7C) and normalize Bax and Bcl-2 activities (Figure 7D and 7E).

Besides blunting the protective impact of *Mettl3* deletion on mitochondrial function, TUNEL assays showed that FCCP attenuated the anti-apoptotic influence of Mettl3 in H/R-exposed HL-1 cardiomyocytes *in vitro* (Figure 7F-7G). Consistently, the CCK-8 assay demonstrated that in the presence of FCCP, cell viability was no longer supported by *Mettl3* ablation (Figure 7H). These findings highlight that in the context of MIRI, the beneficial impact of *Mettl3* deficiency on mitochondrial homeostasis and cardiomyocyte survival primarily rely on negative regulation of the DNA-PKcs/Fis1/mitochondrial fission cascades.

## Discussion

This study provides evidence for the therapeutic potential of *Mettl3* deletion in reperfusion-mediated myocardial dysfunction. At the molecular level, our data indicates that Mettl3 directly interacts with DNA-PKcs to prevent its activation, resulting in decreased Fis1 phosphorylation and attenuated mitochondrial fission. As a consequence, in the setting of MIRI mitochondrial membrane potential and ATP production are sustained, while cardiomyocyte apoptosis is prevented. In addition to protecting mitochondrial homeostasis and exhibiting anti-apoptotic properties, loss of *Mettl3* exerted anti-inflammatory effects in mouse heart tissue following LAD ligation and reperfusion, which also contributed to improved myocardial contractile and relaxation functions. Our data thus provides a novel insight into the intracellular signaling transduction mechanism underlying the cardioprotective influence of *Mettl3* ablation in MIRI. According to current evidence, targeting the DNA-PKcs/Fis1 pathway to normalize mitochondrial fission could be a promising approach for treating MIRI.

The primary finding of our study is that upon both MIRI *in vivo* and H/R *in vitro*, *Mettl3* ablation exerted potent inhibition of mitochondrial fission through suppressing the DNA-PKcs/Fis1 pathway. Actually, several reports have uncovered the regulatory impact of *Mettl3* deficiency on mitochondria in cardiovascular diseases. METTL3, an RNA methyltransferase enzyme, has emerged as a potential player in mitochondrial biologies. Recent studies have suggested that METTL3-mediated RNA methylation may impact various aspects of mitochondrial function and regulation. For example, METTL3 has been shown to influence mitochondrial metabolism by affecting the expression of genes involved in energy production and mitochondrial respiration 13, 42. Additionally, METTL3 has been implicated in mitochondrial dynamics, where its RNA methylation activity may modulate the expression of genes associated with mitochondrial fission and fusion processes 43. Moreover, METTL3 has been found to interact with mitochondrial RNA and potentially regulate the stability and translation of mitochondrial-encoded transcripts 13. These findings highlight the potential involvement of METTL3 in mitochondrial biologies, but further research is needed to fully understand the underlying mechanisms and significance of METTL3 in this context. Importantly, our work elucidated the signaling transduction mechanism by which *Mettl3* deletion inhibits mitochondrial fission, by showing that METTL3 directly interacts with DNA-PKcs and thus prevents MIRI-mediated DNA-PKcs phosphorylation. Previous studies from us 39, 44 and other authors 31, 38 showed that DNA-PKcs activation is induced by phosphorylation, which enhances its kinase activity and leads to phosphorylation of its substrate Fis1. Fis1 phosphorylation increases in turn its affinity for Drp1, therefore stimulating mitochondrial division 39, 45. Although we found that METTL3 is able to bind to DNA-PKcs and thus affect its kinase activity, additional experiments are required to elucidate whether such mechanism accounts for the multiple influence of METTL3 regarding mitochondrial integrity and function in I/R-exposed cardiomyocytes.

Multiple studies have highlighted the significant role of mitochondrial fission in various cardiovascular diseases. Inhibition of mitochondrial fission process 1 has been shown to decrease the progression of heart failure by preserving mitochondrial membrane integrity 46. Additionally, modulation of mitochondrial dynamics through the regulation of the Sirt3/Foxo3a pathway has been associated with a reduction in infarct size following myocardial ischemia-reperfusion injury 47. Bnip3-related mitochondrial fission and mitochondrial autophagy have been implicated in dilated cardiomyopathy 48. In a mouse model of pulmonary arterial hypertension, inhibition of mitochondrial fission through the repression of the ERK/Drp1 pathway has been found to reduce the proliferation and migration of pulmonary arterial smooth muscle cells (PASMCs) 49. Administration of melatonin in diabetic hearts has been shown to prevent Drp1-related mitochondrial fission, leading to a reduction in hyperglycemia-mediated cardiomyocyte death and mitochondrial dysfunction 50. Right ventricular failure induced by pulmonary arterial hypertension has been associated with increased mitochondrial mass and altered mitochondrial metabolism 51. Abnormal mitochondrial fission, particularly related to Drp1 acetylation through an unknown mechanism, has been implicated in lipid overload-mediated cardiomyocyte death and heart dysfunction 52. Furthermore, abnormal mitochondrial fission has been reported to contribute to doxorubicin-related cardiomyocyte death 53. Consistent with these previous findings, our study demonstrated that inhibition of mitochondrial fission through the repression of the DNA-PKcs/Fis1 pathway can reduce cardiac fibrosis following myocardial ischemia-reperfusion injury. Additionally, we identified METTL3 as a novel regulator of mitochondrial fission during cardiac fibrosis. While previous evidence has mostly focused on the role of METTL3 in DNA methylation, our results reveal a novel function of METTL3 in cardiac fibrosis. This discovery opens up new possibilities for therapeutic interventions targeting cardiac fibrosis following myocardial ischemia-reperfusion injury.

In summary, our experiments revealed a novel cardioprotective mechanism by which *Mettl3* ablation attenuates MIRI-related myocardial dysfunction. Three main findings can be concluded from our data: 1) METTL3 directly interacts with DNA-PKcs and thus prevents the activation of DNA-PKcs/Fis1 signaling; 2) *Mettl3* deficiency-mediated inactivation of the DNA-PKcs/Fis1 pathway results in inhibited mitochondrial fission, leading to improved mitochondrial performance and enhanced cardiomyocyte viability; 3) the anti-apoptotic and anti-inflammatory effects of *Mettl3* knockdown may further protect the heart against MIRI. Based on our findings, targeting METTL3 stability shows promise as a valuable therapeutic option for patients at risk of or experiencing MIRI. Besides, our data suggested that the DNA-PKcs/Fis1/mitochondrial fission axis is a relevant target for the design and development of cardioprotective drugs against MIRI.

## Materials and Methods

### Myocardial infarction model and Sirius Red staining

*Mettl3^flox^* mice, *Mettl3* cardiomyocyte knockout (*Mettl3^Cko^*) mice (body weight 23-29 g, aged 11-13 weeks; The Jackson Laboratories) generated as previously described. *Mettl3^flox^* and *Mettl3^Cko^* mice were used to model MIRI 54. ECG was continuously recorded using a PowerLab 16/30 Data Acquisition System. Using a sterile technique, a lateral, minimally invasive intercostal thoracotomy was initially performed 55. Coronary occlusion was maintained for 30 min and reperfusion was induced for 6 h 27. TTC and Evans blue was used to evaluate the infarct size 31. Sirius Red staining was performed as previously described 56.

### Echocardiography

We conducted an evaluation of heart function in *Mettl3^flox^* and *Mettl3^Cko^* mice after MIRI. The mice were induced and maintained under anesthesia using 4-5% and 0.5-1% isoflurane, respectively. Transthoracic echocardiography was performed via a micro-imaging Vevo770 56.

### Electron microscopy

To prepare the tissues for transmission electron microscopy (EM), we first pelleted them by centrifugation. Then, they were fixed overnight in a solution of 1.25% glutaraldehyde in 0.1 M cacodylate buffer. Next, the tissues were dehydrated using ethanol and propylene oxide. Finally, they were embedded in Epon 812 and left overnight to allow for proper embedding. Once the embedding process was complete, the tissues underwent double-contrast staining with uranyl acetate and an aqueous lead solution. Finally, images of the tissues were captured using a CM 10 electron microscope from Philips. 39.

### Cell culture

HL-1 cells, obtained from ScienCell Research Laboratories, were cultured in Dulbecco's Modified Eagle Medium (DMEM) supplemented with 10% fetal bovine serum (FBS) from GIBCO, USA 57. To induce hypoxia/reoxygenation (H/R) injury, the cells were incubated under 100% nitrogen (O_2_ < 1%) at 37 °C for 6 hours. After the hypoxic period, the cultures were returned to normal culture conditions for 12 hours under ambient air and 5% CO2. Lactate dehydrogenase (LDH) levels in both the culture supernatants and within the cells were measured using the Promega CytoTox 96® kit from Promega, Madison, WI, USA. Prior to the H/R injury, HL-1 cells were transfected with control shRNA (sh/ctrl) and *Mettl3* shRNA (*sh/Mettl3*). Additionally, FCCP (10 μM, Cat. No. 370-86-5, Sigma) was added to the media 30 minutes before the H/R injury. Cell viability was assessed using the CCK-8 assay (GK10001, Glpbio, CA, USA) according to the manufacturer's instructions 58.

### Immunofluorescence

The infiltration of neutrophils in myocardial tissue was evaluated by GR-1 immunofluorescence. To this end, fresh heart tissues were embedded in Tissue Tek OCT medium, frozen, and cryosections (8 μm thick) were processed as previously reported 33. Slides were washed with deionized water to remove OCT, fixed in ice-cold acetone, and then incubated with 5% serum 59. Slides were incubated with antibodies against GR-1 (25377, Abcam) at 4°C overnight. Nuclei were counter-stained using DAPI. Images were obtained via an epifluorescence microscope (IX73, OLYMPUS) and quantified using ImageJ software. Results were expressed as the ratio of relative florescence intensity compared with the controls. All quantifications were performed by two observers without knowledge of the identities of samples.

### qPCR

To isolate total RNA from mouse heart or HL-1 cells, TRIzol reagent from Invitrogen was used. After isolation, cDNA synthesis was performed using 1 µg of total RNA and the Transcriptor First Strand cDNA Synthesis Kit from Takara, Dalian, China 60. For quantitative reverse transcription-polymerase chain reaction (RT-qPCR), SYBR Master Mix from Yeasen, Shanghai, China was used according to the manufacturer's instructions 61. The relative expression of the target genes was normalized to the expression of GAPDH. The specific primers used in this study can be found in Supplementary Table S1.

### ELISA

TnT, CK-MB, BNP, and LDH concentrations in serum were measured using a TnT Mouse Uncoated ELISA Kit (LS-F55154-1, LSBio), a CK-MB ELISA kit (LS-F5745-1, LSBio), a BNP ELISA kit (ABIN6954173, Anticorps), and a LDH ELISA kit (ABIN628081, Anticorps) according to the manufacturers' instructions 62. Luminescence was detected with an iMark Microplate Reader (Bio-Rad). The activities of Bcl-2 and Bax in HL-1 cells were measured using a Mouse Bax ELISA Kit (NBP2-69937, Novus) and a Mouse BCL2 / Bcl-2 (Sandwich ELISA) ELISA Kit (LS-F23041, LSBio). The concentration of ATP in HL-1 cells was determined by a Mouse ATP ELISA Kit (MAK190, Sigma). To perform the enzyme-linked immunosorbent assay (ELISA), ELISA plates were coated with a solution of 5 μg/ml methylated bovine serum albumin (Sigma-Aldrich) in phosphate-buffered saline (PBS) and incubated for 12 hours. Purified target proteins, obtained from Sigma-Aldrich and used to construct standard curves, were then added to the plates in PBS and incubated overnight at 4°C 29. After washing, the plates were incubated with a blocking buffer containing 3% bovine serum albumin, 1 mM EDTA, and 0.1% gelatin in PBS for 3 hours. Diluted sera in borate buffered saline (pH 7.4) were added to the plates, followed by the addition of goat anti-mouse IgG(H+L)-alkaline phosphatase from Southern Biotech after washing. The plates were then treated with 4-nitrophenyl phosphate disodium salt hexahydrate (Sigma-Aldrich), and the absorbance was measured at 405 nm. The quantification of target protein contents was determined based on the standard curves 63.

### TUNEL and mitochondrial membrane potential assays and Western blotting

Terminal deoxynucleotidyl transferase biotin-dUTP nick end labeling (TUNEL) was performed using a Plus TUNEL Assay Kit (C10617, Invitrogen). Mitochondrial membrane potential was analyzed using a MITO-ID® membrane potential detection kit (ENZ-51018-K100, AmyJet Scientific Inc., Hubei, China) 64. Western blots were performed as our previously described. 55. DNA-PKc (ab32566, Abcam) and Fis1 (ab229969, Abcam) was used in our study.

### Statistical analyses

The results were presented as mean ± SEM. To compare multiple groups, ANOVA or Welch's ANOVA test, along with Tukey's post hoc test (assuming equal variances), Dunnett's post hoc test, or Tamhane's T2 post hoc test (assuming unequal variances), was conducted. Statistical significance was defined as P < 0.05. SPSS version 28.0 or GraphPad Prism 8.0 was used for all statistical analyses.

## Supplementary Material

Supplementary table.
