# Supplementary material for: METTL3 boosts mitochondrial fission and induces cardiac fibrosis after ischemia/reperfusion injury
Source: Int J Biol Sci. 2024 Jan 1;20(2):433–45. doi: 10.7150/ijbs.87535 (PMC10758110; doi:10.7150/ijbs.87535)
Supplement: Supplementary file 1 — Supplementary table. [file ijbsv20p0433s1.pdf]

Supplemental Table 1: Primer sequences for RT-PCR

Gene	Forward Prime	Reverse Prime
TNFα	5'-AGATGGAGCAACCTAAGGTC-3’	5'-GCAGACCTCGCTGTTCTAGC-3'
IL-6	5'-CAGACTCGCGCCTCTAAGGAGT-3'	5'-GATAGCCGATCCGTCGAA-3'
MMP9	5'-CTTCTGGCGTGTGAGTTTCCA-3'	5'- ACTGCACGGTTGAAGCAAAGA-3'
Gapdh	5′-ACGGCAAATTCAACGGCACAGTCA-3′	5′-TGGGGGCATCGGCAGAAGG-3′
Fis1	5′-GGCTGTCTCCAAGTCCAAATC-3′	5′-GGAGAAAAGGGAAGGCGATG-3′
Drp1	5′-TAGTGGGCAGGGACCTTCTT-3′	5′-TGCTTCAACTCCATTTTCTTCTCC-3′
Mfn2	5′-GCTCCTGAAGGATGACCTCG-3	5′-CGTCTGCATCAGCGTGGACTC-3′
Opa1	5′-CAGTGTTGATGACAGCTCAG-3′	5′-CATCACACACTAGCTTACATTTGC-3′
